# Comparative migration ecology of striped bass and Atlantic sturgeon in the US Southern mid-Atlantic bight flyway

**DOI:** 10.1371/journal.pone.0234442

**Published:** 2020-06-17

**Authors:** Ella R. Rothermel, Matthew T. Balazik, Jessica E. Best, Matthew W. Breece, Dewayne A. Fox, Benjamin I. Gahagan, Danielle E. Haulsee, Amanda L. Higgs, Michael H. P. O’Brien, Matthew J. Oliver, Ian A. Park, David H. Secor

**Affiliations:** 1 Chesapeake Biological Laboratory, University of Maryland Center for Environmental Science, Solomons, Maryland, United States of America; 2 Environmental Lab, USACE Engineer Research and Development Center, Vicksburg, Mississippi, United States of America; 3 Virginia Commonwealth University, Richmond, Virginia, United States of America; 4 Department of Natural Resources, Cornell University, Ithaca, New York, United States of America; 5 Division of Marine Resources, New York State Department of Environmental Conservation, New Paltz, New York, United States of America; 6 College of Earth, Ocean and the Environment, University of Delaware, Lewes, Delaware, United States of America; 7 College of Agriculture, Science, and Technology, Delaware State University, Dover, Delaware, United States of America; 8 Massachusetts Division of Marine Fisheries, Gloucester, Massachusetts, United States of America; 9 Stanford University, Hopkins Marine Station, Pacific Grove, California, United States of America; 10 Delaware Division of Fish & Wildlife, Smyrna, Delaware, United States of America; Institut de Recherche Pour le Developpement, FRANCE

## Abstract

Seasonal migrations are key to the production and persistence of marine fish populations but movements within shelf migration corridors or, “flyways”, are poorly known. Atlantic sturgeon and striped bass, two critical anadromous species, are known for their extensive migrations along the US Mid-Atlantic Bight. Seasonal patterns of habitat selection have been described within spawning rivers, estuaries,and shelf foraging habitats, but information on the location and timing of key coastal migrations is limited. Using a gradient-based array of acoustic telemetry receivers, we compared the seasonal incidence and movement behavior of these species in the near-shelf region of Maryland, USA. Atlantic sturgeon incidence was highest in the spring and fall and tended to be biased toward shallow regions, while striped bass had increased presence during spring and winter months and selected deeper waters. Incidence was transient (mean = ~2 d) for both species with a pattern of increased residency (>2 d) during autumn and winter, particularly for striped bass, with many individuals exhibiting prolonged presence on the outer shelf during winter. Flyways also differed spatially between northern and southern migrations for both species and were related to temperature: striped bass were more likely to occur in cool conditions while Atlantic sturgeon preferred warmer temperatures. Observed timing and spatial distribution within the Mid-Atlantic flyway were dynamic between years and sensitive to climate variables. As shelf ecosystems come under increasing maritime development, gridded telemetry designs represent a feasible approach to provide impact responses within key marine flyways like those that occur within the US Mid-Atlantic Bight.

## Introduction

The ecological and societal services provided by marine fishes are structured by the timing and extent of their migratory behaviors[1–3]. Though broad-scale migration patterns for many marine fishes are well-documented, these are chiefly understood through the context of destinations (e.g. spawning grounds, feeding aggregations). However, behaviors that occur within transit regions are equally important to consider from a fisheries management perspective, as these areas comprise key seasonal habitat in their own right. Similar to avian “flyways”, the networks of migration pathways commonly used by bird species [4,5], coastal fish migration corridors likely function as transit routes while also containing areas where individuals may dwell for extended periods of time to rest or feed [6]. Additionally, like avian migrants, numerous marine species may be seasonally concentrated in shared migration corridors. Though populations and individuals are expected to vary in their specific use of a migration corridor, the multi-species flyway concept emphasizes the broader ecological significance of geospatial routes that may extend over multiple jurisdictional boundaries.

Despite supporting diverse and abundant fisheries, the potential for shelf waters of the US Mid-Atlantic Bight (MAB) to support a multi-species flyway has received little attention. The MAB is connected to multiple crucial estuarine nursery and spawning habitats and is among the most productive coastal systems globally [7]. Though endangered and economically-important taxa seasonally converge within this potential multi-species flyway, patterns of shelf distribution and habitat selection are poorly understood.Further, the current distribution and viability of fish species in the MAB coastal region are likely to change in coming decades. Globally, marine fisheries are threatened by fishing pressure and climate change, both of which will alter species distributions and viability [8–12]. Migratory animals range widely, but their reliance on specific seasonal habitats may increase their vulnerability to anthropogenic impacts [13]. Of particular concern, multiple regions along the US East Coast continental shelf have been leased for the future development of renewable wind energy sites, which are slated to occur in areas that directly overlap with the MAB migration corridor. Construction and maintenance of wind power facilities will have localized impacts, but the widespread extent of development, including other forms energy extraction within species ranges, may fundamentally alter the function of the shelf flyway among individuals and populations.In order for fisheries management to remain effective in this changing environment, increased knowledge of multi-species use of the MAB flyway is needed. The remote nature of the coastal environment has precluded in-depth investigations of such behavioral information in the past, but emerging bio-logging technologies present a valuable opportunity to evaluate the incidence of critical species within the MAB migration corridor [14].

The current state of acoustic telemetry bio-logging along the US East coast provides a unique opportunity to examine fish migrations through coastal shelf waters. In this study, we leverage the robust monitoring capabilities of new receiver technologies and the widespread availability of acoustically-tagged fish in the MAB to understand how this region functions as a multi-species flyway. Specifically, acoustic telemetry was used to evaluate the migration patterns of two model species of management concern: endangered Atlantic sturgeon (*Acipenseroxyrinchusoxyrinchus*; [15,16]) and economically important striped bass (*Moronesaxatilis*). Both species are anadromous (tidal freshwater spawning) with wide-ranging coastal migrations, but differ in their ecology and life history.

Like other fishes of the US Atlantic Coast, broad-scale patterns of movement for Atlantic sturgeon and striped bass have been described through past tagging and mark recapture efforts. In the MAB, individuals generally migrate north in the spring and south in the fall and winter, although evidence of partial migration exists in both species [17–21]. The comparative migration ecology of striped bass and Atlantic sturgeon likely relates to differing foraging and locomotion behaviors, reproductive cycles, and thermal preferences. Atlantic sturgeon are a large, long-lived, anadromous benthivore that range widely in near-shelf waters of the northwest Atlantic (Florida to Quebec). Adult Atlantic sturgeon typically become oceanic residents that make periodic movements into estuaries associated with spawning or straying behaviors. Within MAB coastal environments, Atlantic sturgeon tend to remain in relatively shallow areas close to shore (<50 m depth), with a broader shelf distribution in autumn compared to spring [22–24]. Seasonal concentrations of juveniles and adults also occur near the mouths of inlets and estuaries from North Carolina to Long Island Sound and are hypothesized to be driven by favorable water quality conditions and increased foraging opportunities[23,25,26]. Similar to Atlantic sturgeon, large and mature striped bass (>80 cm) tend to become oceanic migrants [20,27,28], though exceptions do occur: smaller individuals are known to enter coastal waters [29–31]and some adults remain resident in natal estuaries throughout their lives [17,20,32]. Among migratory contingents of striped bass, key destinations during non-spawning phases include northern summer foraging grounds located in coastal Massachusetts [32] and southern overwintering areas along shelf waters near Cape Hatteras, NC [33,34]. In contrast to Atlantic sturgeon, striped bass are highly-mobile pelagic and epi-demersal predators that are often attracted to complex habitats[35–37].

To assess the behavior and habitat preferences of Atlantic sturgeon and striped bass within their coastal flyway, we deployed an array of acoustic telemetry receivers intended to sample the broad, cross-shelf environmental gradients likely to be encountered during transit off the coast of Maryland. Rather than using closely spaced receivers to fully census migrating individuals, the gridded design, focused on relevant spatial gradients, provided better context for evaluating the environmental conditions selected or avoided by Atlantic sturgeon and striped bass during their migratory phase [38]. Similar broad-scale receiver arrangements have been used to examine species behaviors and movement patterns across marine and aquatic habitats globally. Some studies have used true gridded survey designs within smaller areas or enclosed systems (e.g. [38,39]), but many large-scale (> 10 km^2^ coverage using >20 receivers) telemetry applications still employ linear curtains of receivers (e.g. [40–42]) or sampling arrays deployed within focused areas of human concern (e.g. [43–45]). Success of these arrays in gathering crucial baseline data and facilitating management decisions across diverse systems sets a clear precedent for the use of gridded acoustic telemetry arrays in examining current and future migration ecology in the MAB.

Here, we integrate techniques used by previous telemetry studies to maximize monitoring within an area of concern while collecting information across the broader region to gain pertinent ecological information. Though coastal telemetry arrays exist along the US East Coast, studies to date have focused on singular species in specific areas of interest (e.g. [24,46,47]). Here, we utilize a gridded, broad-scale receiver array to facilitate a comparison of the seasonal incidence, behavior, and distribution patterns of the focal species within the MAB flyway.Based on species ecology, we hypothesized that the migratory movements of Atlantic sturgeon and striped bass would differ in terms of transit rate and habitat preference. We anticipated that Atlantic sturgeon would transit more slowly through the shelf region, as they principally forage for benthic prey in soft-bottom habitats similar to those that occur off Maryland’s coast [48–50]. Unlike Atlantic sturgeon, adult striped bass are piscivorous predators that are more likely to occupy pelagic waters. Based on these species’ differences, we hypothesized that striped bass would move more rapidly through this shelf area.

## Materials and methods

### Study site

**Fig 1:**
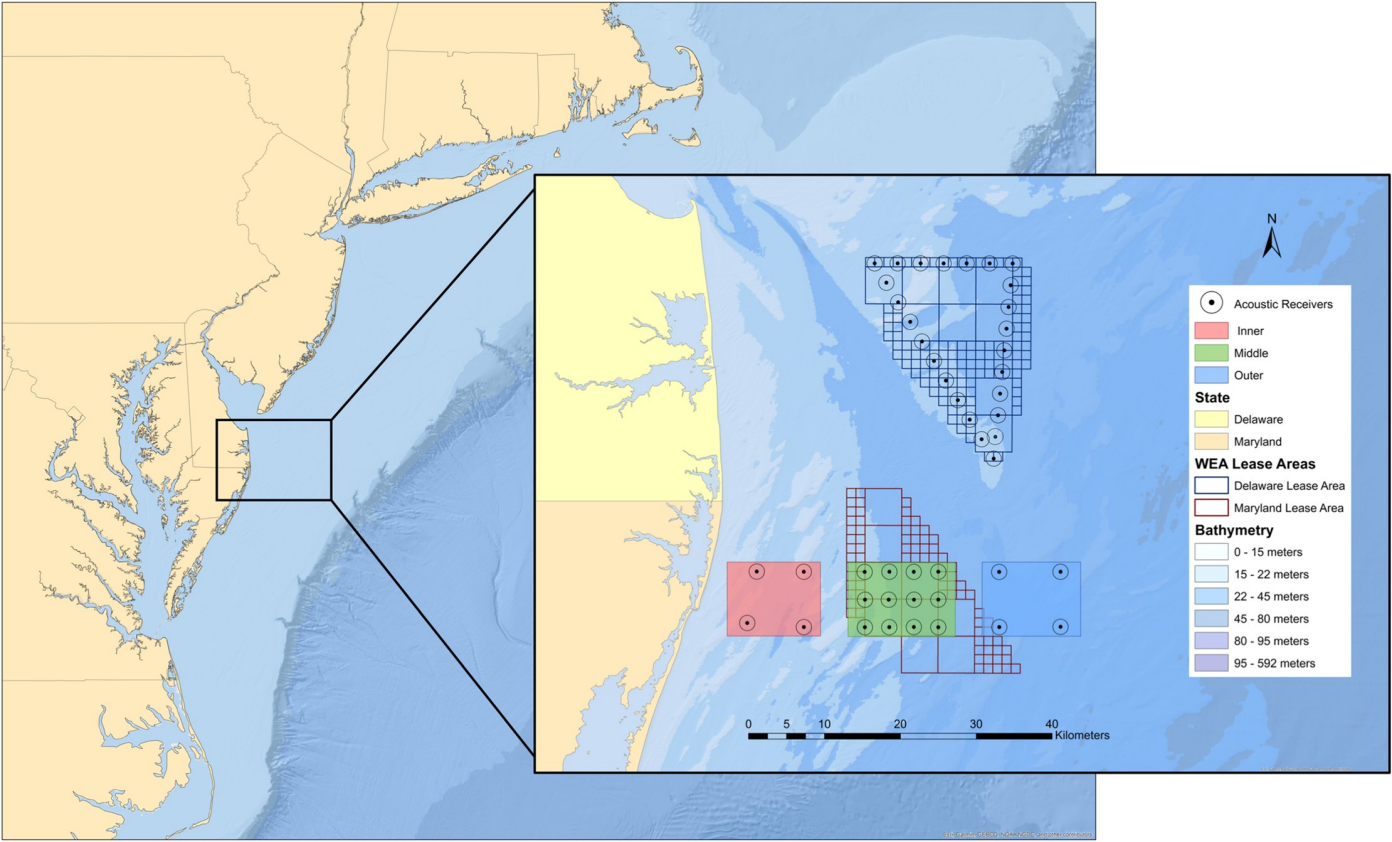
Mid-Atlantic bight study region and acoustic telemetry receiver array design. Delaware (north) and Maryland (south) Wind Energy Areas with respective receiver locations and depth contours are shown. Circles around each receiver represent the expected ~1000 meter maximum detection radius.

The MAB consists of a relatively broad (50–200 km wide) shelf area that stretches from Cape Hatteras, North Carolina to the southern flank of Georges Bank off Massachusetts (Fig 1). Biological dynamics in the MAB are tied to seasonal changes in stratification. During summer, the cessation of strong winds, combined with rapid increases in atmospheric temperature, creates a persistent thermocline that extends over much of the shelf[51,52]. Deeper winter waters maintain relatively constant temperatures even as surface waters warm, resulting in a “cold pool” bounded by warmer near-shelf waters and dense, saltier waters at the shelf break [51,53]. Summer months below the thermocline are therefore characterized by a cross-shelf gradient of decreasing temperature with distance from shore. With the onset of fall, cooling of surface waters, along with wind-driven mixing and storm events that increase bottom water temperatures, destratify the Mid-Atlantic water column [52,54,55]. Shelf water temperatures are thus relatively homogenous throughout the water column during winter months, though a cross-shelf gradient still exists with more-rapid shelf cooling in shallow waters and comparatively warm waters at the outer shelf.South of Hudson Canyon, the MAB is a relatively homogenous and flat seabed habitat, composed primarily of soft sediments [56,57]. The shelf habitat off Maryland exemplifies this pattern; sediments are mainly sandy with low relief and little topographic complexity. However, there are also gravel and mud patches, sand dunes, areas of higher slope, and soft coral habitats that could influence the behavior of fish moving through the area [58].

### Acoustic telemetry array

Movements of acoustically-tagged fish were recorded from November 2016 until December 2018 using a primary array of 20 fixed acoustic-release receivers (VR2AR, 69 kHz; VEMCO-INNOVASEA, Bedford, Nova Scotia, Canada) deployed in a gradient design.Approvals were sought out and received for all detection data used in this manuscript.Here, environmental variables were expected to grade continuously on a spatial and temporal basis; the array design was intended to fully-encompass these cross-shelf gradients by placing receivers at locations to capture this gradient but also target movements through a federal wind farm lease area (MD WEA: Maryland Wind Energy Area). A central and high-density receiver stratum (Middle) was located within the central shelf region. Inshore (Inner) and offshore (Outer) strata of less-densely-distributed receivers were adjoined to this central array (Fig 1). The high density of receivers in the central strata was intended to provide higher-resolution data for baseline movement information within the MD WEA. Receivers were thus positioned across bathymetric and environmental gradients extending over 10–50 km from shore and 10–45 m depth (Fig 1). Based on the *a priori* expectations of a 1000 m maximum detection radius[59], receiver spacing allowed for approximately 50% detection probability in the Middle and 20% detection probabilities in the Inner and Outer strata. The acoustic-release receivers were suspended in the water column 1 m from the seafloor using a 25-cm diameter hard float and two 20.5 kg iron weight plates. Receivers continually recorded detected transmitters, and logged tilt, ambient noise, and bottom temperature on an hourly basis. Data were downloaded approximately every 4 months during maintenance cruises. Supplemental detection data for transit analysis were gathered outside the primary Maryland array through collaborations with University of Delaware and Delaware State University researchers in the Atlantic Cooperative Telemetry (ACT) Network (www.theactnetwork.com).

### Striped bass tagging and available tags

During the period of receiver deployment, >500 striped bass and >1000 Atlantic sturgeon implanted with active transmitters through other studies with different funding and objectives were at large within the MAB and southern New England (www.theactnetwork.com). All tagged fish listed in Table 1 were available for detection over the two years of array deployment, based on their presumed shelf migrations and transmitter battery lifetime (2.5–7 years for striped bass; ≥ 10 years for Atlantic sturgeon). Still, actual availability will be affected by specific migration behaviors, mortality, and the array’s detection efficiency. To further augment striped bass available for detection, an additional 40 large (> 80 cm TL) striped bass were implanted with transmitters to obtain depth-at-transit information for individuals which we expected to undertake coastal movements[28]. A portion of these fish (n = 28) were sampled from a pound net in the lower Potomac River, Point Lookout State Park, MD during April–May 2017 and 2018 (Table 1). Additional tagging of a subset of large striped bass occurred off the coast of Massachusetts during August—October 2017 (Table 1). Fish were surgically implanted with VEMCO®; model V16P-4H-S256 transmittersunder a protocol approved for this study by the University of Maryland Center for Environmental Science IACUC (#F-CBL-17-04), which included use of the anesthetic Aqui-S 20E under a US Fish and Wildlife Service Investigational New Animal Drug permit.

**Table 1:** Summary of tagging information for fish detected in this study.

Species	PI	Institution	N	Tagging location	Period of tag activity	Size range of tagged fish (TL, cm)
AS^a^	D.A. Fox,M.W Breece	DSU^c^	178	Coastal Delaware	2010–2025	160–260
AS	M.T. Balazik	VCU^d^	74	James River	2012–2027	160–240
AS	Others	-	57	New York, Delaware, Maryland, Virginia, South Carolina	-	-
SB^b^	B.I. Gahagan	MA DMF^e^	139	Coastal Massachusetts	2015–2022	NA
SB	D.H. Secor, A.L. Higgs, J.Best	UMCES^f^, NYS DEC^g^	61	Hudson River	2016–2019	70–100
SB	D.H. Secor, B.I. Gahagan	UMCES, MA DMF	15	Coastal Massachusetts	2017–2019	75–85
SB	D.H. Secor	UMCES	13	Potomac River	2017–2019	75–115
SB	I.A. Park	DE DFW^h^	71	Delaware River	2016–2019	57–116
SB	Others	-	16	New England	-	-

^a^AS = Atlantic Sturgeon

^b^SB = Striped Bass

^c^DSU = Delaware State University

^d^VCU = Virginia Commonwealth University.

^e^MA DMF = Massachusetts Division of Marine Fisheries

^f^UMCES = University of Maryland Center for Environmental Science

^g^NYS DEC = New York State Department of Environmental Conservation

^h^DE DFW = Delaware Department of Fish and Wildlife

### Data analysis

Prior to analysis, all acoustic data were filtered to eliminate single detections to help correct for false detection and code collision[60]. Detection data for each transmitter (individual) were compiled to provide incidence at hourly and daily time steps. Temporal patterns in incidence were investigated using generalized autoregressive moving average (GARMA)models to accommodate the non-Gaussian (discrete and zero-inflated) distributions [61]. Two Fourier series of sinusoidal functions, sin(2*πt/d*) and cos(2*πt/d*), where period *d* is one day or one year, and *t* is the hour-of-day or day-of-year, respectively, were added as explanatory variables to determine temporal patterns[62]. Here, day-of-year assesses seasonality while hour-of-day describes diel cycles. Models were fit using the gamlss.util package in R and were selected by Akaike’s Information Criterion (AIC) comparison[63]. Autocorrelation and partial autocorrelation plots were examined for remaining serial dependence in the model residuals and residual plots were used to assess the overall model fit. Daily incidence (no. individual fish d^-1^) was summed by receiver to evaluate broad-scale differences in number of individuals detected between seasons and region (Inner, Middle, Outer). For all analyses, seasons were divided equally and defined as winter (Dec, Jan, Feb), spring (Mar, Apr, May), summer (Jun, Jul, Aug), and autumn (Sep, Oct, Nov). Because daily incidence data were zero-inflated and skewed, non-parametric Kruskal-Wallis one-way analysis of variance and post hoc Dunn’s test with the Bonferroni correction method for multiple comparisons were used. Analyses were conducted using R 3.5.1 [64] and ArcGIS 10.1 (Environmental Systems Research Institute).

Spatial patterns of site (receiver) usage within the gridded array were assessed using the Optimized Hot Spot Analysis tool (Getis-Ord Gi*statistic) in ArcGIS. Hot spot analysis is typically used to determine areas of significant spatial clustering of events over surrounding regions based on the number of observations occurring within defined grid cells (e.g.[65]). Here, the number of individual detections occurring at a receiver were treated as a grid cell covering an assumed maximum 1000 m radius and the ArcGIS tool was used to identify broader areas of individual occurrence hot spots (higher numbers) or cold spots (lower numbers) over all receivers. The distance band for analysis was automatically chosen by the Optimized Hot Spot Analysis software such that spatial clusters of high or low incidence could occur over multiple nearby receivers. Separate analyses were conducted based on the number of unique individuals detected daily at each receiver within each season and over all seasons and years combined.

Single-Parameter Quotient analysis[66] was used to investigate the selection behavior of each species for bottom temperature in each season. Temperatures where fish were detected (daily presence/absence receiver^-1^) were compared to the entire distribution of temperature values that were measured within a seasonal period. Temperature values were binned so that each interval contained a range of 2°C to increase interpretability and to reflect regional and seasonal variability. For each temperature interval, a Quotient index (QI) was calculated as

$${QI}_{i}=\frac{\%Observed\;Detections}{{Env.Var.Freq}_{i}\times 100}$$ (1)

where *i* is *i*-th frequency histogram interval and Env.Var.Freq gives the distribution of daily bottom temperature values recorded in each environmental variable interval for the season. A value of QI = 1 represents even distribution across habitat types, QI>1 indicate preference, and QI<1 indicate avoidance. Selection is operationally defined as greater than expected occupancy in certain habitats based on frequency of habitat availability. Significant deviation from QI = 1.0 was tested through bootstrapping. Confidence intervals (CI) were calculated based on the null hypothesis of a random association between biological and environmental variables. Instances of QI values lying outside of the CI curve indicate significant selection or avoidance.

Indices of residency and transit were calculated from individual data aggregated into broad autumn/winter and spring/summer periods to facilitate comparisons between northern and southern migrations for each species. Residency was calculated using daily incidence data and the V-Track package in R ([67]; c/o Franklin Ecolab, The University of Queensland, St Lucia, Qld, Australia). The RunResidenceExtraction function was used to determine when tagged striped bass and Atlantic sturgeon were within the detection field of a given receiver [47,68]. Each detection event for a tagged fish was initiated when the individual first moved into the detection field and was recorded two times. Detection events were then terminated when the tag was detected at a different receiver or if no new detections were recorded for 12 hours, a more conservative measure of how long telemetered species might be present within receiver detection radii without being detected than the standard 24-hour cutoff used in the V-Track package [67]. Residence events were summed for each fish and each migration season and reported as hours detected. Cumulative unique days detected for each individual per season were also calculated to provide a comparative, coarse measure of residence. Differences in residence periods between species and seasons were compared using Wilcoxon rank sum tests.

Speed of transit was estimated for both species based on transit from the MD telemetry array to a nearby array in shelf waters off the Delaware Bay, an array centered in the Delaware Wind Energy lease area deployed from February 2017–2019 (Fig 1). Similar to the MD array, Delaware receivers were moored to the bottom but were suspended slightly higher in the water column (approximately 4 m off the seafloor). Transit events were defined as directed one-way movements. Each transit event was classified as north or south and rate of transit was calculated as the distance (m) between the two receivers divided by the amount of time (s) between detections. Any movements that were longer than one month in duration were excluded from analysis to limit skewing of the data due to prolonged stopovers or missed detections. Differences in swimming speed between direction (north vs. south) and species were evaluated using Wilcoxon rank sum tests. Transit rates were further tested for differences according to life history characteristics using generalized linear mixed effect models (GLMMs) in the R package lme4 [69]. Rate of transit was the dependent variable with season as a categorical variable and body size at tagging (TL; total length in cm) as a continuous covariate. As all fish were assumed to be adults, individuals were not expected to increase substantially in length over the two years of this study. Unique individual (tag code) was added as a random effect in the models to account for repeated measures. The importance of season and TL at tagging for transit rate was investigated by comparing models with the null model (random effects only, without fixed effects).

## Results

### Detections

A total of 352 Atlantic sturgeon and 315striped bass tagged in diverse MAB locations were detected by the coastal MD array between November 2016 and December 2018 (Table 1). Nearly half of the Atlantic sturgeon were counted as present only once during the study (174 fish, 49%); 34% and 14% were detected in two and three separate migration seasons (autumn/winter and spring/summer of any year), respectively. Of the remaining 9 fish that occurred in >3 migration seasons over multiple years, most were tagged off the coast of Delaware (n = 6). Acoustically-tagged striped bass had an overall higher seasonal fidelity to the array, with 41%, 34%, and 25% of individuals detected across ≥ 3, 2, and 1 migration seasons.

### Temporal patterns of distribution

**Fig 2:**
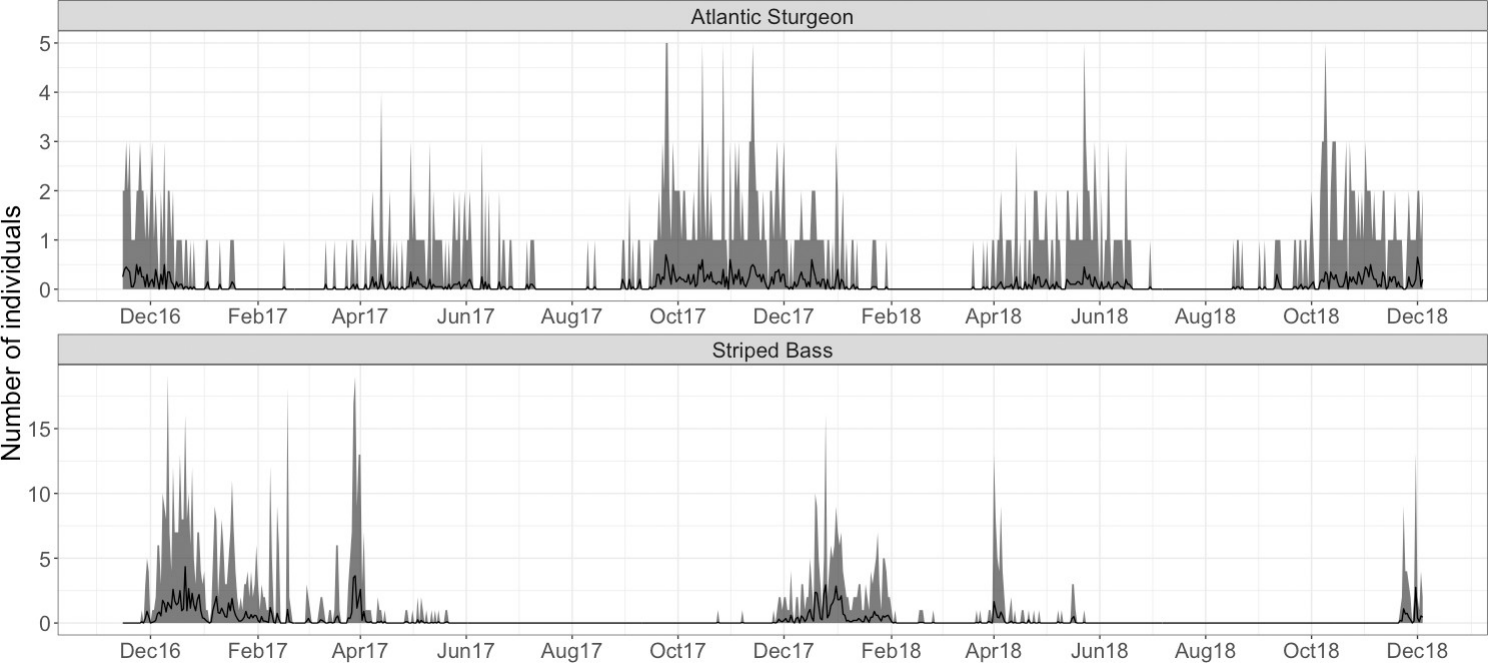
Number of unique individual atlantic sturgeon and striped bass recorded daily. Total daily incidence is based on summed unique individual detections at each receiver. Gray shading represents the minimum and maximum values of incidence across the array. Black lines show the mean number of individuals detected across the array. Note the differing scales on the y-axis for each species.

**Fig 3:**
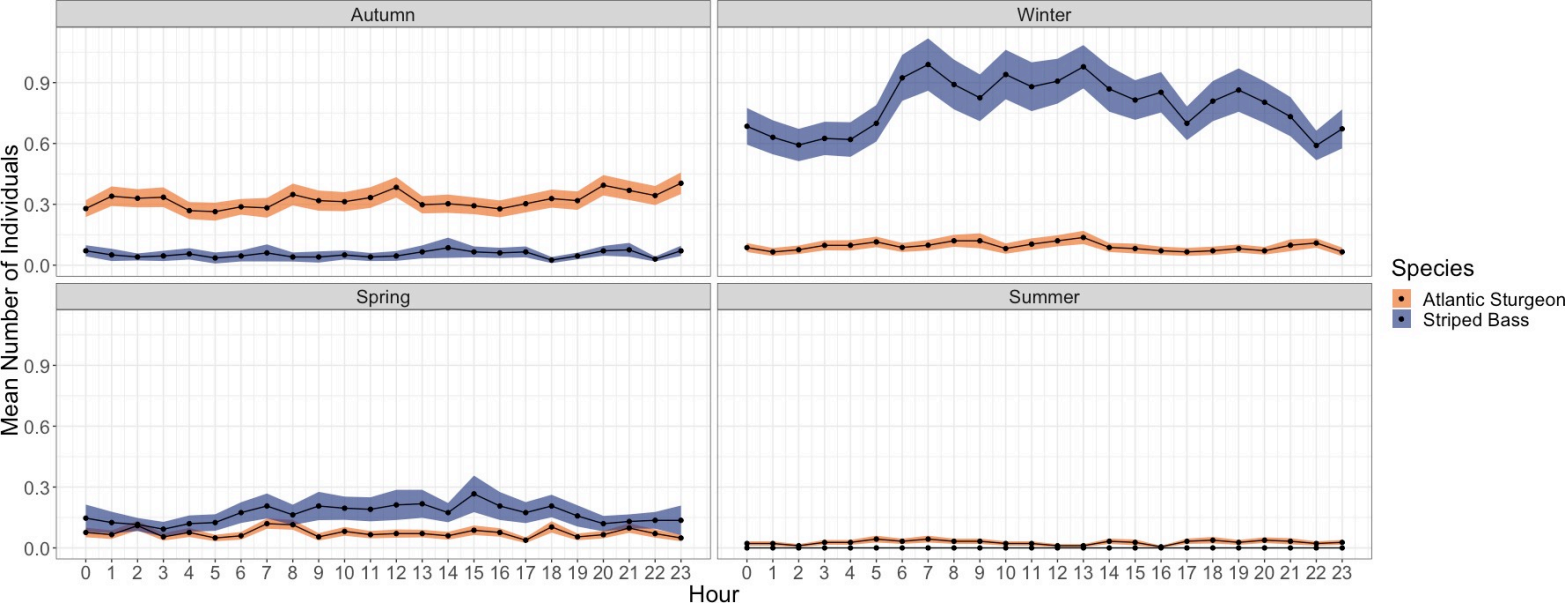
Hourly mean number of unique individual fish detected seasonally. Seasons are aggregated across years of the study (November 2016-December 2018). Shaded bands represent the standard error.

Seasonal components (sine and cosine transformations of day-of-year) were retained as highly significant in the final GARMA models for both species (Table 2). Atlantic sturgeon occurred over broad periods during early spring to early summer and early autumn to early winter each year (Fig 2), with very few detections during late-summer or late-winter months. Compared to Atlantic sturgeon, striped bass had a higher mean number of individuals detected on each receiver per day (Fig 2). Additionally, striped bass exhibited more-sporadic but concentrated seasonal incidence; greater numbers of individuals occurred December-February and early April both years. Striped bass were consistently absent from the array across summer and autumn months. Hour-of-day was retained as a significant predictor in the final GARMA model for striped bass incidence, but not for Atlantic sturgeon (Table 2). Striped bass were more likely to be detected within the array during daylight hours, especially during winter months (Fig 3). Atlantic sturgeon lacked a diel pattern among seasons (Fig 3). Although cyclic patterns were identified in GARMA model residuals for both species, these likely reflected the exceptionally zero-inflated distribution of individual hourly detections. Residuals were, however, normally-distributed and lacked temporal autocorrelation, indicating that models adequately fit the daily and seasonal detection patterns.

**Table 2:** Parameter estimates and best distributions for generalized autoregressive moving average (GARMA) models.

Parameter	Atlantic Sturgeon	Striped Bass
**Distribution**	Zero-inflated poisson	Negative binomial
**β**^**a**^ **intercept**	-756.608*** (46.632)	-557.196*** (84.026)
**β sine hour**	-	477.956*** (36.099)
**β cosine hour**	-	-248.956*** (50.301)
**β sine day**	249.633*** (22.337)	0.149*** (0.043)
**β cosine day**	233.368*** (26.547)	-0.103* (0.041)
**θ**^**b**^_**1**_	0.491*** (0.022)	-0.453*** (0.018)
**θ**_**2**_	-0.437*** (0.026)	-0.156*** (0.017)
**θ**_**3**_	-0.11*** (0.023)	-0.06*** (0.016)
**φ**^**c**^_**1**_	0.155	1.0
**φ**_**2**_	0.843	-

Parameter inclusion and best distributions chosen based on AIC rankings. Sine/cosine hour parameters signify daily patterns while sine/cosine day parameters represent seasonal patterns. Standard errors are included in parentheses where applicable and significance of parameters are indicated by asterisks (0 ‘***’ 0.001 ‘**’ 0.01 ‘*’ 0.05 ‘.’ 0.1 ‘ ‘ 1.).

^a^β = Regression coefficients

^b^θ = Autoregressive parameters

^c^φ = moving average parameters

There were significant differences in the number of individuals detected seasonally for each species (Table 3). The z-scores of pairwise comparisons using Dunn’s post hoc test showed that more Atlantic sturgeon were detected during autumn compared to all other seasons (autumn incidence higher than spring: *p*< 0.001; summer: *p*< 0.001; winter: *p*< 0.01)and that individual incidence was greater during the winter than the summer (winter incidence higher than summer: *p* = 0.002). Incidence of Atlantic sturgeon did not differ between spring and summer or winter and spring (spring incidence higher than summer: *p* = 0.205; winter: *p* = 0.420). Striped bass pairwise comparisons showed significant differences in incidence between all seasons except between spring and winter (spring incidence higher than winter: *p* = 0.364). Individual striped bass incidence was highest in the winter (winter incidence higher than autumn: *p*< 0.001; summer: *p*< 0.001) and lowest in the summer (summer incidence lower than autumn:*p* = 0.003; spring: *p*< 0.001).

**Table 3:** Kruskal-Wallis (K-W) and Dunn’s post-hoc test results for numbers of individuals detected between seasons and strata.

Species	K-W		Dunn’s test			K-W		Dunn’s test	
	*X*^*2*^	*p*	*z*	*p*		*X*^*2*^	*p*	*z*	*p*
**Atlantic Sturgeon**									
**Season**	42.85	<0.001*			**Stratum**	52.27	<0.001*		
Autumn-Spring			4.257	<0.001*	Inner-Middle			3.234	0.002*
Autumn-Summer			6.253	<0.001*	Inner-Outer			7.092	<0.001*
Autumn-Winter			3.120	0.006*	Middle-Outer			5.452	<0.001*
Spring-Summer			1.822	0.205					
Spring-Winter			-1.473	0.420					
Summer-Winter			-3.471	0.002*					
**Striped Bass**									
**Season**	86.20	<0.001*			**Stratum**	8.949	0.01*		
Autumn-Spring			-3.689	<0.001*	Inner-Middle			-2.863	0.006*
Autumn-Summer			3.266	0.003*	Inner-Outer			-1.068	0.429
Autumn-Winter			-5.856	<0.001*	Middle-Outer			1.555	0.198
Spring-Summer			6.349	<0.001*					
Spring-Winter			-1.549	0.364					
Summer-Winter			-8.504	<0.001*					

K-W tests assess differences in mean number of individuals detected and Dunn’s test results show significance of pairwise season and stratum categorical factors. Asterisks indicate statistical significance of differences in number of individuals detected.

Cross-shelf strata differences were evident across all seasons (Table 3). Pairwise comparisons of number of individual Atlantic sturgeon were significant for all strata with the Inner stratum exhibiting higher average incidence than the Middle (*p* = 0.002) and Outer (*p*< 0.001) regions, and the Middle stratum having higher individual incidence than the Outer stratum (*p*< 0.001). In contrast, striped bass incidence only varied significantly between the Middle and Inner strata; in this case, more individuals were detected in the Middle region over the study period (*p* = 0.006).

### Environmental drivers of occurrence

**Fig 4:**
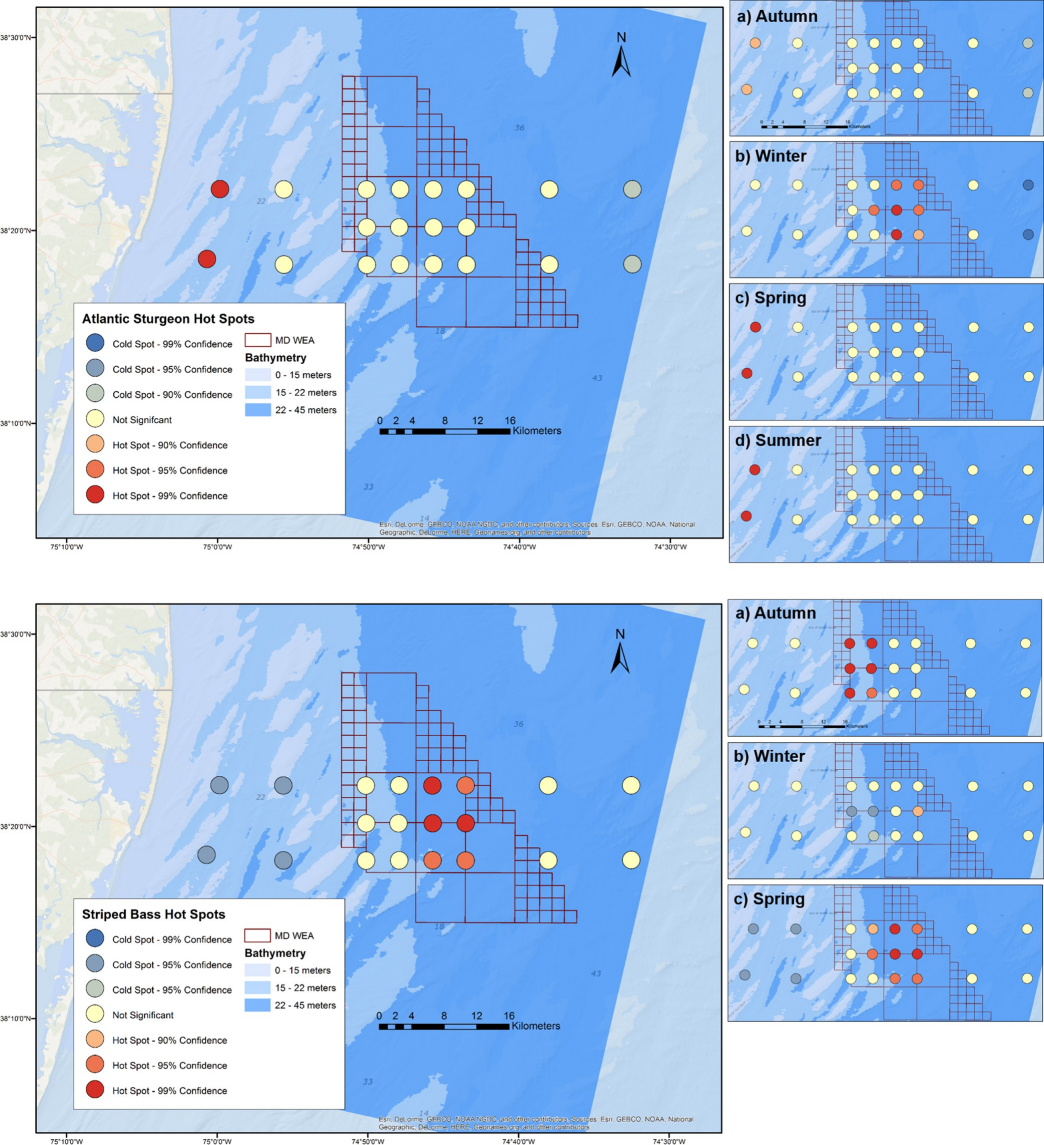
Hot spots of species occurrence across the acoustic receiver array. Results reflect annual (left) and seasonal (insets, right) numbers of individual Atlantic sturgeon (top) and striped bass (bottom) detected per receiver.

The two receivers closest to shore were a hot spot for Atlantic sturgeon, especially during spring and summer seasons (Fig 4). This hot spot diminished during the autumn, with simultaneous evidence for a cold spot (90% confidence, *p*< 0.1) at the deepest Outer stratum receivers in the same season. During winter, there was an area of increased clustering for Atlantic sturgeon in the deeper section of the Middle stratum and a significant cold spot (99% confidence, *p*< 0.01) at the deepest sites. Striped bass detection hot spots were only identified within the Middle region. However, clustering of individual incidence occurred at shallower depths during the autumn compared to winter and spring. No striped bass were detected during summer months.

**Fig 5:**
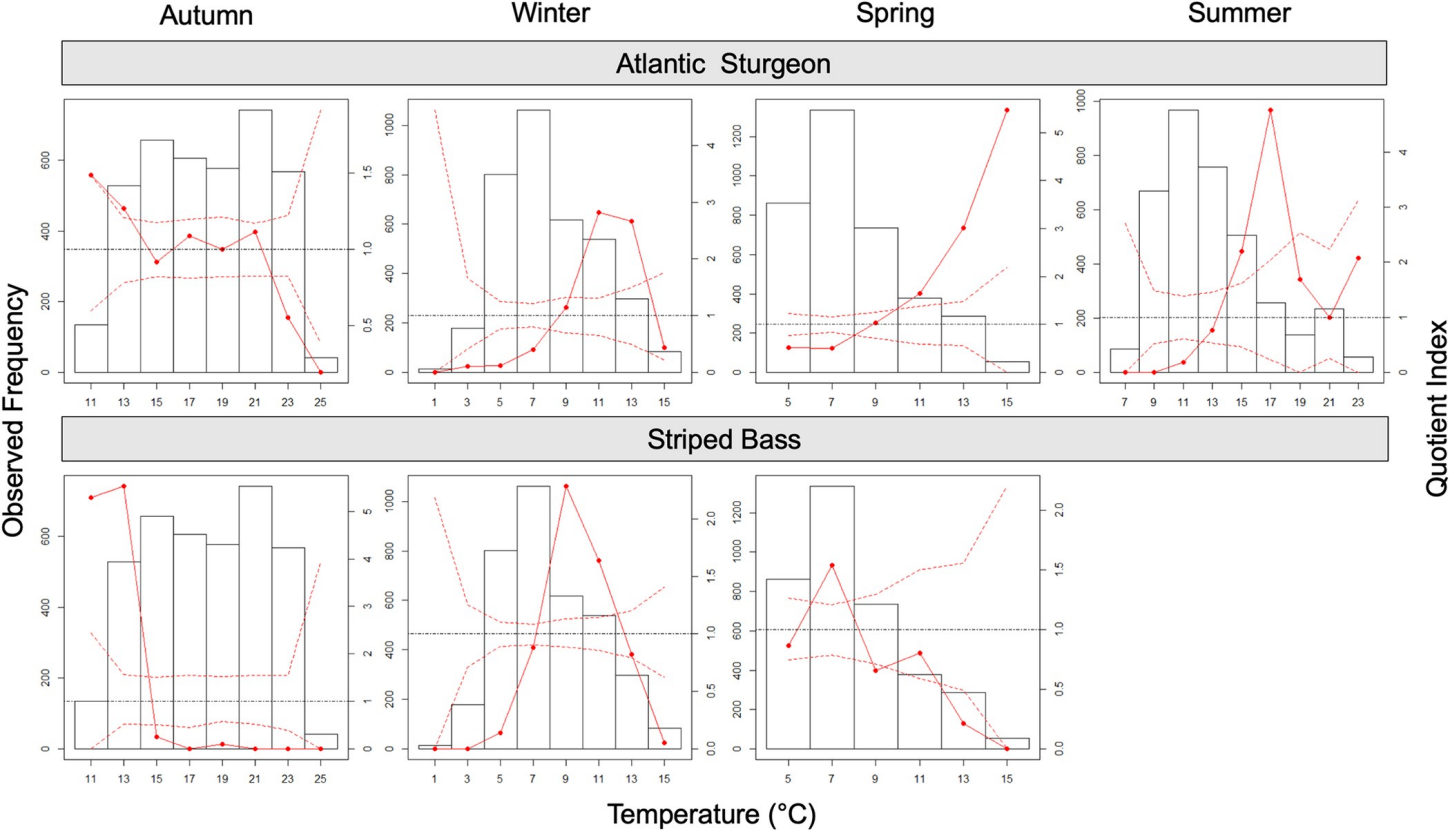
Seasonal temperature preference of study species. The quotient analysis results assess the distribution of Atlantic sturgeon (top) and striped bass (bottom) presence/absence data to receiver-recorded bottom temperature categories across seasons.Each plot shows the observed quotient index (QI) curve (solid red line), its confidence interval (dashed red lines), and the frequency histogram of bottom temperature. Points within the confidence band represent tolerance for temperature conditions while points above the band suggest selection and points below the band indicate avoidance. The dotted black line in each plot indicates the value QI  =  1.

Occupancy of warmer bottom temperatures by Atlantic sturgeon and cooler bottom temperatures by striped bass was a key difference between the two species as they migrated through the study area. Quotient analysis showed that across all seasons, Atlantic sturgeon typically occurred at relatively high bottom temperatures between 9–22°C, as recorded by receivers (Fig 5). Indeed, there was little evidence for temperature preference by Atlantic sturgeon during autumn, when most bottom temperatures were warm and between 12–22°C. During winter and spring, when temperatures were cooler, Atlantic sturgeon significantly selected temperatures >11°C. Warmer temperature preference was also apparent during summer, when Atlantic sturgeon tolerated all temperatures >13°C but occurred more often in the 15–18°C range. In contrast, striped bass significantly avoided temperatures higher than 15°C across seasons. During autumn, striped bass only occurred in the coolest available temperatures between 11–14°C. Selection was again relatively narrow in winter months but occurred between 9–13°C, with apparent tolerance for temperatures just outside this range and avoidance of more extreme seasonal bottom temperatures above 14°C or below 7°C. Striped bass broadly tolerated temperatures between 5–12°C in spring months with preference occurring within the 7–8°C temperature bin. Temperatures higher than 13°C were avoided by striped bass during this season, but wider confidence bands (as a result of low sample size) limit this inference.

### Individual migration characteristics

Degree of residency tended to be low for both Atlantic sturgeon and striped bass; average cumulative time spent in the detection radius of receivers per migration season was less than 4 hours for both species (Atlantic sturgeon: mean ± SE = 3.04 ± 0.26 hr; striped bass: 3.25 ± 0.13 hr). Total number of unique days detected for each species were also relatively low across migration seasons (Atlantic sturgeon: mean ± SE = 1.6 ± 0.04 d; striped bass: 2.55 ± 0.05 d). Differences in residency were statistically significant, with striped bass occurring for more hours and days than Atlantic sturgeon (Wilcoxon rank sum test, hours: *p*< 0.001; days: *p*< 0.001). Striped bass were also detected for more hours and days during autumn/winter months compared to spring/summer months (hours: *p*< 0.001; days: *p*< 0.001). Like striped bass, Atlantic sturgeon were detected for more days on average during autumn/winter months (*p*<0.001) but hourly presence did not differ between migration seasons (*p* = 0.09).

**Fig 6:**
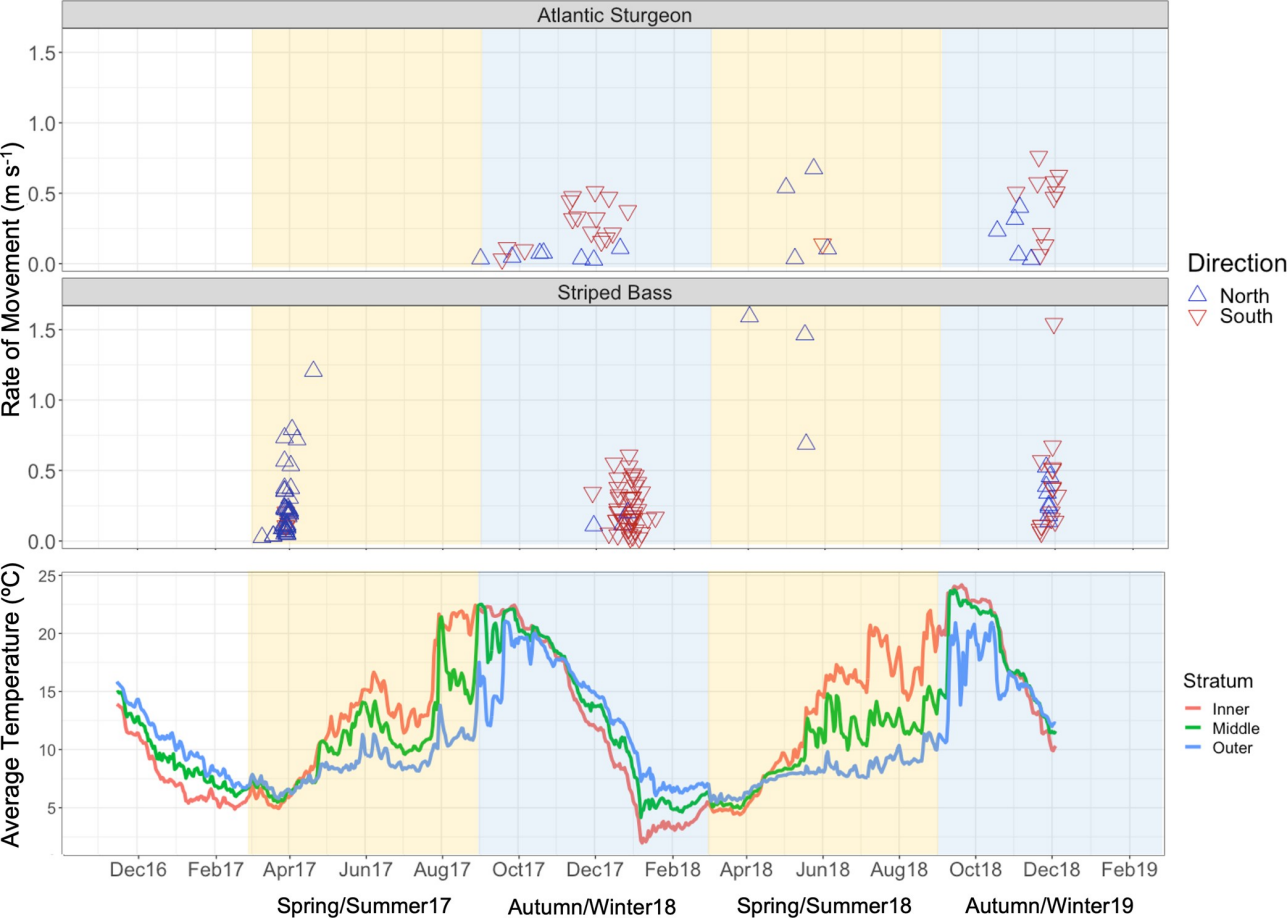
Summary of transit information by species from November 2016—December 2018. Shading denotes separate migration seasons (Spring/Summer = yellow, Autumn/Winter = blue). Plots include direction and rate of transit for each study species (top two panels) and average bottom temperature recorded by receivers (bottom panel).

Serial detections of Atlantic sturgeon and striped bass between the coastal MD and DE arrays mostly occurred in the expected direction of movement: south in autumn/winter and north in spring/summer (Fig 6). There were occasional instances where individuals made both north and south transits within a migration season (Atlantic sturgeon autumn/winter: 10 of 26 individuals, Atlantic sturgeon spring/summer: 1 of 5 individuals, striped bass autumn/winter: 10 of 50 individuals, striped bass spring/summer: 3 of 41 individuals). Although Atlantic sturgeon were detected in both arrays during spring/summer 2017, all sequential detections were separated by more than a month and so were excluded from analysis to limit spurious detections across seasons or between distant receivers. For striped bass, sequential detections between arrays were noticeably reduced during spring of 2018, in which telemetered fish were detected more often in the MD array than the DE array (159 detections in MD, 39 detections in DE; Fig 6). Of these MD detections, 94% occurred within the Outer stratum (140/149 total MD array detections).

Transit rate did not differ between species (Wilcoxon rank sum test, *p* = 0.89). Atlantic sturgeon swimming speed varied depending on the direction of transit (*p* = 0.005), with more rapid southerly movements across seasons (north: mean ± SE = 0.18 ± 0.05 m s^-1^; south: 0.33 ± 0.04 m s^-1^). For striped bass, during autumn/winter, mean transit rate in the southerly direction was 0.28 m s^-1^ ± 0.03 SE and during spring/summer, mean transit rate in the northerly direction was 0.31 m s^-1^ ± 0.06 SE. Although the fastest observed transit rates for striped bass (>0.5 m s^-1^) tended to occur in the northern direction during spring, speed was similar between the two directions ofmovement (*p* = 0.80). The maximum observed striped bass transit rates of 1.5 m s^-1^ would translate to about two body lengths s^-1^. The null model for Atlantic sturgeon transit rate was effectively similar to single-term models (Δ AIC < 2), indicating that season and TL at tagging did not affect transit rate between individuals (Table 4). However, all generalized linear mixed models for striped bass that contained season and TL as fixed effects were better ranked in terms of AIC score than the null model. TL at tagging and season thus influenced speed of striped bass movement (Table 4; TL-only model: TL *p* = 0.01; season-only model: season *p*< 0.01); however, only TL was found to significantly affect transit rate in the full model, with larger fish being more likely to transit faster between arrays (Full model: season *p* = 0.14, TL *p*< 0.01).

**Table 4:** Transit rate model results.

Model	df^b^	AIC^c^	Δ AIC	LogLik^d^
**Atlantic Sturgeon**				
m s^-1^ ~ (1 | Tag)	3	57.86	0.00	-25.932
m s^-1^ ~ Season + (1 | Tag)	4	59.46	1.60	-25.727
m s^-1^ ~ TL^a^ + (1 | Tag)	4	59.60	1.74	-25.799
m s^-1^ ~ TL + Season + (1 | Tag)	5	61.19	3.33	-25.595
**Striped Bass**				
m s^-1^ ~ TL + Season + (1 | Tag)	5	151.34	0.00	-70.669
m s^-1^ ~ TL + (1 | Tag)	4	151.46	0.12	-71.732
m s^-1^ ~ Season + (1 | Tag)	4	152.12	0.78	-72.061
m s^-1^ ~ (1 | Tag)	3	153.37	2.03	-73.683

Generalized linear mixed model results and model factors for considered Atlantic sturgeon and striped bass transit models including Tag ID as a random effect (1 | Tag) to account for repeated measures. The best-ranked models are at the top under each species heading.

^a^TL = Total length at tagging in cm

^b^df = Degrees of freedom

^c^AIC = Akaike’s Information Criterion

^d^LogLik = Log Likelihood

## Discussion

### Comparative migration ecology

In this study, acoustic telemetry and data sharing allowed us to evaluate and compare the behavior of two species within their migratory flyway. Striped bass and Atlantic sturgeon were transient off the coast of Maryland but differed in their seasonal distribution and use of shelf habitat. Rapid movements through the study area occurred for both species, with evidence that larger striped bass transited at a faster rate than smaller individuals. Relatively few telemetered fish were detected for periods > 24 hr and detection histories were characterized by long periods of absence, particularly for striped bass. It is important to note that residency, as recorded here, is conservative due to the limited spatial detection range of receivers. Still, multi-day periods of incidence were observed for both species during autumn and winter. Although striped bass were relatively transient during spring months, individuals were often present for 3 or more days during winter. These results contradict our original hypothesis that the coastal stock of striped bass would rapidly transit through the MAB and suggest some individuals may use this region for overwintering habitat.

When present, Atlantic sturgeon and striped bass preferred distinct habitat conditions; Atlantic sturgeon tended to occur in warmer near-shelf waters while striped bass were more likely to select cooler and deeper areas. However, habitat preference differed seasonally with Atlantic sturgeon having a wider distribution during their fall migration and striped bass selecting deeper waters as near-shelf temperatures rapidly cooled in winter. These seasonal patterns appeared to reflect broader cross-shelf distributional shifts related to depth and temperature gradients rather than selection for specific benthic characteristics.

The observed patterns of Atlantic sturgeon presence were largely consistent with known aspects of species migration patterns. Tagging and bycatch records in the MAB shelf region have reported the highest numbers of Atlantic sturgeon captures occurring in the spring and fall [22,70]. Atlantic sturgeon were generally absent from the late spring through early fall, when they are occupying riverine spawning and nearshore foraging habitats [22,71]. During the winter, Atlantic sturgeon may be inhabiting relatively warmer habitats to the south, near Virginia and Cape Hatteras, where they have been shown to aggregate[22,25,26,72]. Broader shelf distributions during autumn than during spring and summer have also been observed in landings records, surveys, and electronic tagging studies [22–25].

In contrast to our original hypothesis that Atlantic sturgeon would slowly transit the study region, movements were relatively quick, with only a few instances (n = 15) of seasonal residence ≥ 24 hr. These transit rates may indicate a lack of favorable conditions available for Atlantic sturgeon in our study region, although the identified window of temperature selection between 9–22°C is well within the known range of thermal tolerance for this species [73,74]. The lack of apparent residency by Atlantic sturgeon may instead relate to this area serving mainly as a transit route between northern spawning and nearshore spring/summer feeding grounds and southern winter habitat. Atlantic sturgeon in the coastal ocean are known to concentrate around the mouths of inlets and estuaries in spring, summer, and fall[23,26]. Within these regions, sturgeon have been found to associate with river plumes or sandy and muddy substrates that may offer increased foraging opportunities [25,49,75,76]. Faster southerly transits may further support the tendency to rapidly exit the study area in favor of southern winter aggregation areas as temperatures in the northern MAB become unsuitably cool. This is not to say however, that Atlantic sturgeon did not forage during their occupancy of the study site, where substrate and benthic productivity should support this activity [77–79].

Patterns of striped bass occurrence also aligned with established seasonal migrations of south in the fall and north in the spring, but revealed unique patterns of oceanic incidence in winter and in deeper waters. Striped bass are known to overwinter in the nearshore waters off Cape Hatteras[80–82], but other portions of the migratory contingent appear to winter in the shelf region as far north as Cape Cod, Massachusetts [17,28,34,83]. Our results show that individuals move to areas > 50 km from the coast and occupy the region for an extended period in winter. Peaks in frequency of occurrence during daylight hours may further support the use of Maryland’s shelf waters as overwintering habitat for this species; striped bass are predominantly visual predators and could be increasing activity on a diel basis to locate and capture prey. Though striped bass were associated with a relatively narrow range of temperatures, we did identify a lower temperature threshold of 5°C, indicating that striped bass avoid the coldest oceanic temperatures that occur in the near-shelf region during winter. Other fish species in the Northwest Atlantic undertake similar cross-shelf distributional shifts during winter, including black sea bass(*Centropristisstriata*), fluke (*Paralichthysdentatus*), and scup (*Stenotomuschrysops*)[84,85]. Like these species, striped bass (and Atlantic sturgeon, to a lesser extent) may select warmer outer shelf waters over the cooler near-shelf waters of the MAB in winter.

Striped bass movement behavior during spring supported our original hypothesis of rapid transit through the MAB shelf region. The highest rates of transit tended to occur in a northerly direction in the spring, corresponding with northward movement toward Delaware and Hudson River spawning areas or summer foraging grounds located off the coast of Massachusetts [86–88]. Spring transits > 1 m s^-1^, the fastest estimated speeds of movement recorded for striped bass in this study, would translate to roughly 1–1.5 body lengths s^-1^ for an 80 cm TL striped bass. Although these speeds are well below maximum sustained swimming speeds of 2.9–3.3 body lengths s^-1^ for striped bass [89], they are greater than mean southern transits, which were closer to 0.3 m s^-1^, or 1/3 body lengths s^-1^. Still, uniform directionality was not always observed and sequential detections were not consistent during spring 2018, leading to non-significance in transit speed between the MD and DE arrays. Other telemetry studies have found highly variable rates of transit during spring; some transit intervals between the Delaware Bay and Massachusetts were as rapid as 9 days (transits of 1.6 m s^-1^over a 500 km straight-line distance) while other fish stopped for hours to days in bays and estuaries along their migration route [90]. Although striped bass exhibited directed movement in our study area, this does not preclude extended stopovers in the areas like the Delaware Bay, New Jersey estuaries, or Long Island Sound during the spring, which have been recorded in the past [90,91,92]. Similar to our findings, migration intervals calculated by Kneebone et al.[90] were, on average, shorter for northward movements of striped bass tagged in Massachusetts. Similarly, Callihan et al. [93]found that striped bass spawning in the Roanoke River showed directed movements (mean = 0.68 m s^-1^; maximum = 0.92 m s^-1^) to northern oceanic regions. Results collectively indicate that striped bass emigrate relatively quickly from southern overwintering and spawning regions, likely motivated by warming temperatures.

### Study design

The gridded, cross-shelf gradient design, not previously employed in Atlantic telemetry studies, provided useful inferences in the comparative migration behavior of striped bass and Atlantic sturgeon. Coastal or marine telemetry studies tend to utilize linear receiver gates to assess passage of acoustically-tagged individuals. While gates deployed across geographic bottlenecks provide a high degree of certainty regarding fish presence or absence, these arrangements can also have substantial spatial bias [94,95]. Studies employing gridded receiver arrays offer a more statistically-robust approach for sampling the environment while simultaneously permitting observation over a larger range of habitat types [38]. Here, we used a gradient-based extension of the gridded approach to better incorporate hypothesized continuous (gradient) drivers of fish migration. To cover a large shelf region, we undertook a sampling rather than a census tactic, the latter requiring ≥100% receiver detection ranges. Though density of individuals detected in the MD array seemed relatively low (1–2 individuals per receiver daily), and sustained occurrence was brief, the intentionally dispersed array design likely underestimated the number of individuals present and the amount of time they spent in the area. Ultimately, detections of hundreds of tagged Atlantic sturgeon and striped bass from diverse tagging origins in the study region highlights the broader importance of the MAB as a migratory corridor.

By gathering information across biologically relevant spatial (shelf-wide) and temporal (multi-seasonal) scales, the study design lent itself to the analysis of species habitat selection [96,97]. For instance, the adjacent DE array recorded far fewer striped bass detections during spring of 2018, despite consisting of more closely-spaced receivers that were better-positioned to census individuals transiting the proposed Delaware WEA. The tradeoff of favoring increased receiver line efficiency over broader spatial shelf coverage meant that the DE array was not able to capture a potential migration behavior change in striped bass, likely because it did not extend far enough into deeper shelf waters. Relatively cooler temperatures during the 2018 migration season may have caused striped bass to move faster or farther offshore. Striped bass were almost exclusively detected at Outer receiver sites during this migration season, which contrasted their occurrence across Middle and Outer locations during the spring of 2017. This difference in distribution suggests that the migration corridor for striped bass shifted further toward the outer shelf in 2018, into a region that was not monitored by the DEarray. Although wider receiver spacing and variable detection range may have inflated our assumed absences and led to lower apparent site fidelity, we maintain that these tradeoffs were necessary to understand this segment of the MAB flyway. Still, other designs (i.e., arrangements of receivers) might hold greater advantage depending on whether the purpose was to detect single or multiple species. For instance, a gradient design for Atlantic sturgeon would be focused more inshore than one for striped bass. Additionally, the latitudinal arrangement of arrays should be revisited against monitoring goals. Here, transit rates within the MD array were not feasible and required ancillary data from the DE WEA array.

Though our receiver grid comprised a large swath of available cross-shelf habitat, this area represents a small portion of the entire range inhabited by migratory striped bass and Atlantic sturgeon. Our results thus describe a restricted window along an extended migration corridor and inferences may not be applicable to other latitudes of the MAB. Similarly, migration cues likely occur outside the study area. For example, though interannual differences in the wintertime occurrence and cross-shelf distribution for striped bass could be related to measured habitat variables within the study site, the timing and speed of migration probably depends on conditions and seasonal cues occurring in other shelf regions or spawning tributaries such as the Hudson River and Chesapeake Bay. Migratory behavior is often considered preemptive in that individuals will depart areas before they become unfavorable [98]. In the case of estuaries like the Chesapeake Bay, striped bass will emigrate before temperatures become too warm and metabolically demanding, particularly for large individuals > 90 cm TL [99,100]. However, local habitat attributes still likely influenced patterns of occurrence; striped bass may have transited through the area using deeper offshore waters during winter and spring of 2018 because they were avoiding excessively cold nearshore temperatures or reacting to changes in availability of prey.

### Implications

Climate change is now altering marine species distributions in unpredictable ways [8–10]. Already, poleward shifts have been observed in some northwest Atlantic coastal fishes[11,12]. Changes in population response may be particularly complex for species like striped bass and Atlantic sturgeon that range widely and tolerate a broad range of habitat conditions but also exhibit natal homing to particular estuaries. Based on our results, both species may experience an expansion of preferred temperature conditions on the MAB shelf under a warming climate, especially striped bass during winter months. However, natal homing for these species to specific estuaries both north and south of the study area means that climate will drive more-complex spatial and temporal migration changes rather than wholesale population shifts in range. For instance, though Canadian populations of Atlantic sturgeon and striped bass are not well represented in the MAB, these groups show specific adaptations to their physical and thermal northern environments. In contrast to our study, Atlantic sturgeon migrate pelagically through the Minas passage and forage in crepuscular patterns during summer in the Minas Basin of Canada [50,101]. Overwintering striped bass in the same area, near the northern extreme of their range, occasionally tolerate temperatures <1°C and may have broader temperature tolerance compared to more southern populations [102,103]. Given such considerable differences in latitudinal ecology, population structure will be a key consideration in evaluating migration behavior as climate changes. Although natal origin of telemetered fish was not considered in this study, similar array designs could be employed to assess population-specific shifts in flyway habitat use under changing shelf conditions. Population segment specific preferences may infer wholesale population shifts in response to climate change without the need for decade long studies.

The development of offshore renewable energy infrastructure could also alter coastal migration behaviors within the coming decade. Currently, multiple wind farm installation sites have been leased along the US East coast in areas that coincide with migration corridors. The critical nature of the shelf flyway, combined with the presence of individuals over extended seasonal time periods, means Atlantic sturgeon and striped bass should be a concern for wind energy development. Wind tower construction and site maintenance activities such as pile-driving, amplified vessel traffic, increased sedimentation, or altered electromagnetic fields caused by power cables could result in physiological stress or avoidance of the area by marine species [104–109]. Other renewable energy developments, such as tidal turbine installations at the northern extent of species ranges, may pose additional threats in the form of collisions [101,103].However, within the relatively featureless MAB, added structure from wind turbines may provide habitat throughout the water column and introduce refuge or forage resources for both demersal and pelagic fishes [110–112]. Despite the lack of baseline data prior to construction, recent meta-analyses show that European wind farms harbor higher abundance and diversity of fish species compared to adjacent reference sites[113]. While benthic feeders such as Atlantic sturgeon may experience a reduction in available habitat due to wind turbine construction, pelagic species like striped bass may be particularly likely to dwell or pursue prey around such novel structure. Mesopelagic fish, striped bass potentially among them, have been found to aggregate around currently non-active tidal turbine platforms in the Bay of Fundy [114]. Although benthivores may show less behavioral change in response to wind turbine construction, an altered MAB shelf environment may thus create novel stopover points for previously transient species that could affect overall migration ecology differently for Atlantic sturgeon and striped bass. New traditions of residency or fidelity will be a management concern worthy of investigation in both species, as these could eventually shift the extent and timing of species-human interactions.

Coordinated telemetry arrays using gradient sampling designs, along with increased cooperative data-sharing and analysis, will serve to expand current knowledge on the migration ecology of marine fishes within coastal flyway corridors. Further, as population ranges change owing to climate forcing and other influences, transboundary collaborations will be important in monitoring for such changes. Establishing comprehensive baselines will also allow managers and stakeholders to evaluate future impacts of climate change and offshore wind farm development.
